# Genetic Variation and Heritability of Sensory and Artisan Bread Traits in a Set of SRW Wheat Breeding Lines

**DOI:** 10.3390/foods12132617

**Published:** 2023-07-06

**Authors:** Maria P. Castellari, Senay Simsek, Jae-Bom Ohm, Robert Perry, Hanna J. Poffenbarger, Timothy D. Phillips, Krista L. Jacobsen, David A. Van Sanford

**Affiliations:** 1 Department of Plant and Soil Sciences, University of Kentucky, Lexington, KY 40546, USA; paula.castellari@uky.edu (M.P.C.); hanna.poffenbarger@uky.edu (H.J.P.); tim.phillips@uky.edu (T.D.P.); 2 Department of Food Science and Whistler Center for Carbohydrate Research, Purdue University, West Lafayette, IN 47907, USA; ssimsek@purdue.edu; 3 USDA-ARS, Edward T. Schafer Agricultural Research Center, Cereal Crops Research Unit, Hard Spring and Durum Wheat Quality Laboratory, Fargo, ND 58108, USA; jae.ohm@usda.gov; 4 Department of Dietetics and Human Nutrition, University of Kentucky, Lexington, KY 40546, USA; rrperr2@uky.edu; 5 Department of Horticulture, University of Kentucky, Lexington, KY 40546, USA; krista.jacobsen@uky.edu

**Keywords:** local and regional food systems, loaf volume, bread-making quality, genomic prediction, protein concentration

## Abstract

Focus on local food production and supply chains has heightened in recent years, as evidenced and amplified by the COVID-19 pandemic. This study aimed to assess the suitability of soft red winter (SRW) wheat breeding lines for local artisan bakers interested in locally sourced, strong gluten wheat for bread. Seventy-six genotyped SRW wheat breeding lines were milled into whole wheat flour and baked into small loaves. Bread aroma, flavor, and texture were evaluated by a sensory panel, and bread quality traits, including sedimentation volume, dough extensibility, and loaf volume, were measured to estimate heritability. SE-HPLC was performed on white flour, and breeding lines were characterized for different protein fraction ratios. Heritability of loaf volume was moderately high (h^2^ = 0.68), while heritability of sedimentation volume, a much easier trait to measure, was slightly lower (h^2^ = 0.55). Certain protein fraction ratios strongly related to loaf volume had high heritability (h^2^ = 0.7). Even though only a moderate heritability estimate of dough extensibility was found in our study, high positive correlations were found between this parameter and sedimentation volume (r = 0.6) and loaf volume (r = 0.53). This low-input and highly repeatable parameter could be useful to estimate dough functionality characteristics. Flavor and texture heritability estimates ranged from 0.16 to 0.37, and the heritability estimate of aroma was not significantly different from zero. However, the sensorial characteristics were significantly correlated with each other, suggesting that we might be able to select indirectly for aroma by selecting for flavor or texture characteristics. From a genome-wide association study (GWAS), we identified six SNPs (single nucleotide polymorphisms) associated with loaf volume that could be useful in breeding for this trait. Producing high-quality strong gluten flour in our high rainfall environment is a challenge, but it provides local growers and end users with a value-added opportunity.

## 1. Introduction

Wheat (*Triticum aestivum* L.) is the most widely grown cereal in the world. As a source of both carbohydrates and protein, it provides the majority of daily calories in many diets [1]. Around 700 million tons of wheat are produced worldwide every year [2]. In Kentucky, wheat is grown on approximately 216,000 hectares with an average yield of 4.9 tons ha^−1^ over the last 10 years [3]. In addition, wheat is a suitable crop to grow as a double-crop under no-tillage systems in a crop rotation following corn (*Zea mays* L.) and preceding soybean (*Glycine max* (L.) Merr.). Furthermore, wheat provides significant agroecological services, such as winter cover, to reduce soil erosion and improve soil health and economic revenue at a different time of the year than summer crops, improving farmers’ cash flow.

Kentucky’s soft red winter (SRW) wheat grain is typically sold as a commodity to millers and grain elevators and produces a weak-gluten flour that is used by the industry for cakes, pastries, and cookies. At the same time, an interest in identifying value-added markets for Kentucky-grown wheat is growing rapidly as the industry and consumers are more eager to source ingredients locally for their baked goods. Furthermore, it is expected that farmers would receive increased prices by supplying high-quality grain; thus, there is an economic potential to be exploited by gaining access to those specialized markets. Therefore, when evaluating the quality characteristics of wheat grown in Kentucky, it is of great interest to understand what needs to be carried out next to supply the growing local demand.

Specialized, value-added markets for premium quality products have already been developed for other crops that are usually grown and sold as commodities. For instance, a study by Rekik et al. [4] demonstrated that achieving desirable traits in coffee can unlock a price premium potential, improving farmers’ incomes. Another study involving maize concluded that, although the current protocol among nearly all large-scale bourbon distilleries is to utilize commodity yellow dent hybrid corn, variation in flavor and alcohol yield still occurs between hybrids and can be targeted by characterizing their diverse organoleptic profiles [5]. Unfortunately, there is an expressed concern that modern wheat selection programs have narrowed the genetic base of newer wheat varieties by the propagation of successful, high-yielding varieties that are genetically related. In this scenario, introducing aroma and flavor quality as a new breeding criterion could contribute to broadening the genetic diversity of modern bread wheat.

According to a previous study on wheat [6], aroma and flavor parameters have become valuable criteria in consumer selection. However, although bread aroma is one of the first characteristics perceived by consumers, it is not typically assessed as a quality parameter by the milling or baking industry [2]. According to Chang et al. [7], the quality of bread can be judged based on loaf volume, texture, color, and flavor. In addition, Laidig et al. [8] highlighted that the baking quality of wheat is mainly determined by protein concentration and protein quality, but the proportion of the variability in loaf volume that can be explained by differences in protein concentration is inconsistent between studies. Unfortunately, the contribution that wheat flour makes to bread flavor has not been so well described, although some studies noted that components found in wheat flour are likely contributors to overall bread flavor [9].

Gluten strength depends on grain protein concentration and composition [10]. Wheat storage proteins consist of monomeric gliadins and polymeric glutenins. Gliadins affect dough extensibility, while glutenins are associated with dough elasticity [11]. Glutenins are differentiated by their molecular weight as high molecular weight subunits (HMW-GS) and low molecular weight subunits (LMW-GS). The HMW:LMW ratio is used as a predictor of a cultivar’s bread-making quality [12]. In addition, the development of methods to evaluate the protein size distribution, such as size-exclusion HPLC (SE-HPLC), has contributed to an understanding of the role of the different protein fractions and their association with bread-baking quality [13]. For instance, Gupta et al. [14] reported a significant correlation between the percentage of SDS-unextractable polymeric protein (%UPP) and extensograph maximum resistance. Singh et al. [13] reported high and positive correlation coefficients between the relative quantity of glutenin obtained through SE-HPLC analysis and loaf volume, extensograph dough resistance and extensibility, and mixograph peak development time of flour from 15 wheat cultivars with diverse bread-making quality. Another study used the SE-HPLC methodology and multivariate analyses to develop models that were able to categorize hard and soft white wheat with high precision [15].

In the context of creating value-added products, establishing a standard method of performing sensory analysis of bread might help increase the value of wheat flour as well as the interest of consumers, considering that sensory attributes of food allow the consumer to quantify quality [16]. A sensory evaluation involves collecting responses as food is tasted and can be used in cereal science to determine how much a person likes a baked good or to describe the intensity of the sensory properties of the product [17]. Some studies have indicated that it is possible to perceive differences in various grain sub-products due to genotypic variability using sensory panel evaluations. Starr et al. [6] were able to distinguish between different wheat varieties which were prepared as cooked grains. Using a sensory panel to evaluate unaged-whiskey, Arnold et al. [5] found differences attributable to the variety used for its distillation, the terroir, and their interaction. Moreover, Herb et al. [18] found differences in beer sensory descriptors when comparing barley genotypes.

In addition to describing sensorial characteristics, research is needed to examine the genetic basis and the heritability of these quality traits to be able to incorporate them into a breeding program. Research on heritability and the genetic basis of dough functionality traits is more commonly found in the literature [19,20,21] than similar research on sensory analysis. Unfortunately, breeding for sensorial traits is hindered by the time-consuming evaluation activity, which can reduce the number of lines that a breeding program is able to analyze. In this sense, the study from Longin et al. [2] highlighted the key role that genomic prediction can play in these kinds of traits by training a predictive model with a subset of lines and determining the performance of the remaining genotypes with molecular marker data. Moreover, being able to identify proxies of quality characteristics that reduce the amount of time spent in baking would be of great value and could help wheat breeders incorporate new evaluation parameters to supply farmers and, ultimately, the industry with high-baking-quality wheat varieties.

Given this background, the goals of the present work were to: (i) quantify and characterize genetic variation in sensory and dough functionality characteristics among breeding lines from the University of Kentucky (UK) Wheat Breeding Program, (ii) estimate the heritability of these traits to understand if we can incorporate them as selection criteria in a breeding program, and (iii) analyze the relationship between sensory parameters, dough functionality parameters, and protein fractions. The results of this research should help to identify breeding lines that have desirable baking qualities in combination with superior agronomic traits as well as the capacity to be grown in Kentucky and the mid-south region and sold to local artisan millers and bakers.

## 2. Materials and Methods

### 2.1. Grain Source

For this study, 76 genotyped lines from the UK Wheat Breeding Program were evaluated from two growing seasons (seasons 2019–2020 and 2020–2021). The first year of replicated, multi-location testing for these genotypes was 2020 as part of the Advanced Yield Trial Stage, and the following year these lines were advanced to the Max Trial Stage so that we had 2 years of multi-location yield and agronomic trait characterization and 2 years of evaluation for Fusarium head blight resistance.

The lines were planted in October and harvested in July for both seasons at Spindletop Research Farm near Lexington, KY (38°7′37.81″ N, 84°29′44.85″ W), where the soil type was Maury silt loam (a fine, mixed, active, mesic Typic Paleudalf; [22]). Weather data can be found in Supplemental Table S1. Management was in accordance with common practices of the region for fertilization, weeds, pests, and disease control [23]. Conventional practices call for nitrogen (N) applications at Feekes growth stages 3 and 5. In addition, given that we were planning to bake bread, a late N application was supplied at heading (Feekes growth stage 10.5; [24]) to boost grain protein level. The source of N for the three applications was urea, broadcast and hand-applied at a rate of 35, 70, and 35 kg N ha^−1^ at the three respective growth stages. Once the grain was cleaned after harvest, samples were stored in a freezer at −20 °C until milling and baking.

Protein concentration and predicted kernel hardness were measured using near-infrared reflectance spectrometry (NIR) from whole grain samples with a DA 7250 NIR analyzer (Perten Instruments, Hägersten, Sweden).

### 2.2. SDS Sedimentation Volume

As a predictive measure of protein quality, a sodium dodecyl sulfate (SDS) sedimentation test was performed following the AACC Method 56–70 [25] with minor modifications. This method measures the relative gluten strength in whole wheat meal and does not require sieving. It is useful for screening plant breeding materials due to the low amount of meal needed.

For this procedure, 5 g of whole wheat meal were obtained with a Cyclone sample mill (UDY, Fort Collins, CO, USA) equipped with a 1 mm sieve. One gram of meal was placed in a 25 mL glass graduated cylinder, and 4 mL of distilled water were added. Each cylinder was vortexed at maximum speed for 3 s. After a 5 min rest, the cylinders were vortexed again for 3 s and left to sit for 5 more minutes. This procedure was repeated one more time for a total of 3 iterations. Subsequently, 12 mL of SDS-lactic acid solution were added, and cylinders were inverted 10 times on a tube rocker. Tubes were placed in an upright position and left to settle for 15 min before the sedimentation volume was recorded as milliliters of the sediment column. Duplicated evaluations were conducted for each sample, and mean values were used for data analysis for each season.

The SDS-lactic acid solution was prepared daily by adding 20 g SDS powder and 20.8 mL of refluxed 0.1% (*v*/*v*) lactic acid to 1000 mL distilled water. The solution was stirred for 30 min until these substances were thoroughly dissolved and placed into a bottle-top dispenser.

### 2.3. Extraction and SE- HPLC Analysis of Protein Fractions

The flour protein was extracted for size-exclusion HPLC (SE-HPLC) as described by [14] with minor modifications [26,27]. White flour samples were obtained with a Brabender Quadrumat Junior mill (Brabender^®^ GmbH & Co. KG, Duisburg, Germany). The SDS-extractable fraction was obtained with 1 mL of 1% SDS and 0.1 M sodium phosphate buffer (pH 6.9). The residue was resuspended in 1 mL of the extraction buffer in order to solubilize SDS unextractable proteins and sonicated in a probe-type sonicator (Sonic Dismembrator 100; Fisher Scientific, Waltham, MA, USA). SE-HPLC was performed using an Agilent 1100 Series (Agilent Technologies, Santa Clara, CA, USA). The SDS-extractable and SDS-unextractable protein fractions were analyzed individually on a narrow-bore size exclusion column (Yarra 3µm SEC S4000, 300 × 4.6 mm, Phenomenex, Torrance, CA, USA) with a precolumn in-line filter assembly (0.2 μm, 0.125″ dia. SST Frit, Analytical Scientific Instrument US Inc., Richmond, CA, USA) and a guard cartridge (BioSep SEC S4000) [27]. Duplicated evaluations were conducted for each sample, and mean values were used for data analysis for each season.

Absorbance data from SE-HPLC chromatograms of protein extracts were interpolated and analyzed with an in-house program that was developed using MATLAB (version 6, The MathWorks, Natick, MA, USA) [15,27,28]. The SE-HPLC profiles were divided into four fractions (F1–F4) for flour samples for both SDS extractable and unextractable fractions. Primary components constituting individual fractions were high molecular weight polymeric proteins (F1), low molecular weight glutenin polymers (F2), gliadins (F3), albumin, globulins, and protein hydrolysates (F4). The total was equal to SDS extractable plus SDS unextractable data.

Different ratios were calculated from the absorbance area raw data, such as gliadin to glutenin (F3 to F1 + F2 fractions), HMW:LMW (F1 to F2 ratio), or UPP:TPP (SDS unextractable polymeric protein to total polymeric protein). For this study, absorbance area percentage (A%) data were preferred over absorbance area (AA, expressed in mAU*min) since AA values represent a quantitative variation in protein eluted at a given moment, thus affected by the total protein content, while A% is the percentage of AA at a given retention time interval over total AA and represents the variation in protein compositions [15].

### 2.4. Bread Baking

For each tasting session, grain from five breeding lines plus a control (‘Edison’—hard white spring wheat) was milled on a Mockmill 100 Stone Grain Mill (Mockmill USA, Fairfield, IA, USA). Two hundred grams of whole-wheat flour were obtained and used for baking small loaves of bread. The bread was made following a commonly used 70% hydration bread recipe. Small amounts of yeast (1 g) were used in order to limit the yeast flavor and odor influence in the final product, with a 20 h fermentation process at 3 °C. Measurements taken from the baked bread included loaf height, weight, and volume, and loaf density was calculated from loaf weight and volume.

Before baking, a 50 g sample was separated from the dough to perform the windowpane test to evaluate dough extensibility. The windowpane test consisted of rolling the sample of dough into a ball and manually stretching it between the fingers to form a thin layer without breaking it. The capacity of the dough to be gently stretched without tearing it was scored on a 1 to 7 scale, where 1 is the lowest resistance to stretching, and 7 is the highest. This methodology is typically used by bakers as a qualitative assessment of gluten development and sufficiency of kneading or stretching and folding.

### 2.5. Sensory Panel Evaluation

The sensory panel evaluation consisted of 4 to 6 evaluators that assessed each bread sample. The sensory panel judged the overall aroma, flavor, and crumb and crust texture of each sample on a scale from 1 (dislike very much) to 7 (like very much) with unit intervals. Evaluations of bread samples from the grain of the 2019–2020 season were conducted between January and May of 2021, usually once a week in groups of 6 samples. Evaluations of samples made with the grain from season 2020–2021 were conducted between August and December of that same year, also once a week with 6 samples of bread each week. The evaluator panel remained the same between the two seasons in order to reduce the variability of the evaluations.

### 2.6. Data Analysis

Overall sensorial characteristics (aroma, flavor, and texture parameters from the sensory panel evaluation) as well as SDS volume, protein concentration, and predicted kernel hardness were analyzed using JMP^®^ Version 16.0.0 (SAS Institute Inc., Cary, NC, USA) with the following generalized linear model (GLM) in order to determine genotypic differences and estimate heritability:

(1) $$y_{ijk}=\mu +a_{i}+r_{j}:a_{i}+g_{k}+a_{i}g_{k}+e_{ijk},$$

where $y_{ijk}$ was the observed trait for the *i*th year, in the *j*th replication of the *k*th genotype, μ the general mean, $a_{i}$ the effect of the *i*th year, $r_{j}:a_{i}$ the effect of the *j*th replication within the *i*th year, $g_{k}$ the effect of the *k*th genotype, $a_{i}g_{k}$ the interaction effect of the *i*th year and the *k*th genotype, and $e_{ijk}$ the residual error. In the case of sensory evaluation parameters, the evaluators were considered the replications.

Heritability and 90% confidence intervals were estimated from the mean squares (MS) obtained from the analysis of variance as in [29]:

(2) $$h^{2}=1-\frac{MS\left[genotype\times year\right]}{MS\left[genotype\right]},$$

(3) $$UL=1-{\left[\left(\frac{MS\left[genotype\right]}{MS\left[genotype\times year\right]}\right)\times F_{1-\frac{\alpha }{2};df1,df2}\right]}^{-1},$$

(4) $$LL=1-{\left[\left(\frac{MS\left[genotype\right]}{MS\left[genotype\times year\right]}\right)\times F_{\frac{\alpha }{2};df1,df2}\right]}^{-1},$$

where UL is the upper limit and LL the lower limit of the confidence interval, and F_1−*α*/2:*df*1,*df*2_ indicates the value from the F distribution such that the probability of exceeding this value is 1 − *α*/2. F_*α*/2:*df*1,*df*2_ is the analogous statistic. When replication within the year was not feasible, heritability estimates were calculated using the genotypic variance ($\sigma_{g}^{2}$) and error variance ($\sigma_{e}^{2}$) components, and year as a replicate (r), as in [2]:

(5) $$h^{2}=\frac{\sigma_{g}^{2}}{\left[\sigma_{g}^{2}+\left(\frac{\sigma_{e}^{2}}{r}\right)\right]}.$$

Such was the case for loaf volume, loaf density, loaf height, dough extensibility, flour protein concentration, and flour protein fractions from SE-HPLC.

In addition, a principal component analysis (PCA) was performed based on flavor, aroma, and texture parameters, as well as dough functionality traits collected for each genotype across the two seasons. With the individual variables scaled and centered, Pearson’s correlation coefficient between traits was calculated.

A second principal component analysis was performed using the protein fractions data from SE-HPLC, SDS sedimentation volume, predicted kernel hardness, and flour and grain protein concentration. The ultimate goal of performing a separated PCA with these variables was to obtain the principal component scores to be used to fit a regression model (PCR) with measured loaf volume [30,31].

### 2.7. SNP Calling and Genome-Wide Association Study (GWAS)

SNP genotype calling was carried out as in [32]. DNA from leaf samples was extracted using the Sbeadex plant kit from BioSearch Technologies, and genotyping by sequencing (GBS) was performed following the protocol from Poland and Rife [33]. The final number of retained SNPs was 12,808 after filtering by missing data (≤50%), minor allele frequency (≥5%), and heterozygous calls per marker locus (≤10%).

GWAS was performed using data from four “environments”: harvest year 2020 (ENV1), harvest year 2021 (ENV2), average data from both years (ENV3), and BLUPs (ENV4). We fitted three models, BLINK, FarmCPU, and GLM, using GAPIT v3.1.0 package in *R* [34]. The cutoff critical *p*-value was set at 1/n, where n was the number of SNPs, and only those SNPs with false discovery rate (FDR) adjusted *p*-value less than 0.1 were considered as significant trait-associated QTL. To establish more rigorous criteria, we required that SNPs should be declared significant in at least two environments or using two GWAS models to be considered significant for our results. Specific information about each model can be found in [34].

## 3. Results

**Figure 1:**
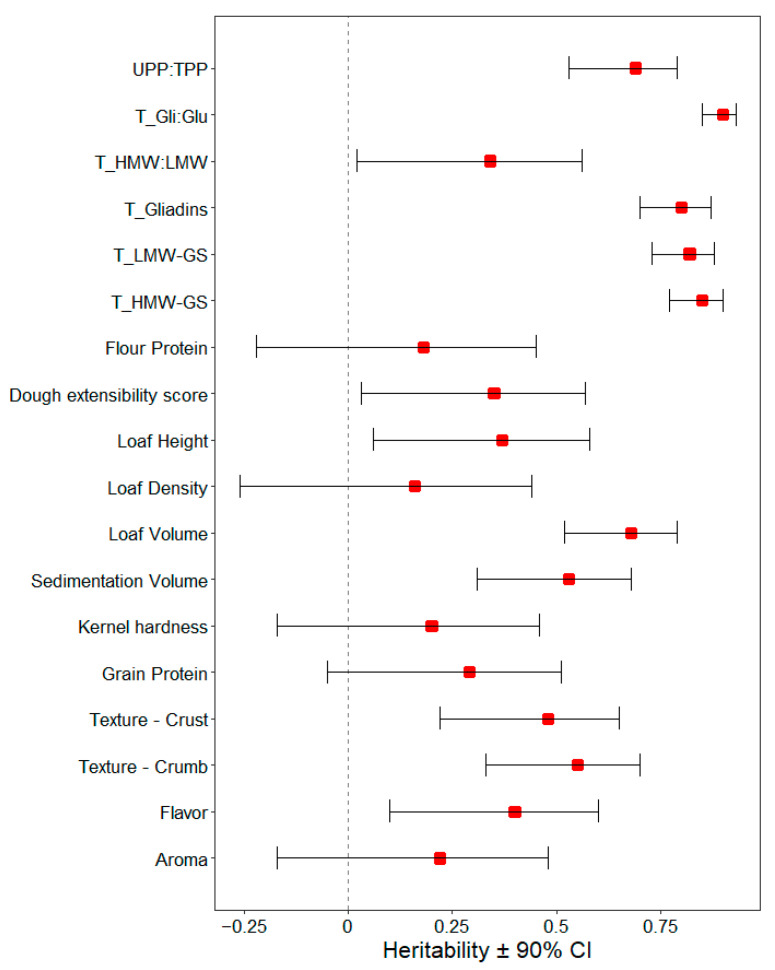
Heritability estimates calculated from mean squares for all quality parameters under study and flour protein fractions from SE-HPLC. Red squares represent the broad-sense heritability estimate (h^2^), and horizontal error bars represent 90% confidence intervals (CI). The vertical dashed line represents h^2^ = 0. “T_”: total SDS extractable and SDS unextractable protein fractions; UPP:TPP: SDS unextractable polymeric protein to total polymeric protein ratio.

The summary statistics and ANOVA components used to calculate heritability estimates of the quality parameters are shown in Table 1. Heritability estimates with 90% confidence intervals are shown in Figure 1. High heritability estimates (*h*^2^ = from 0.68 to 0.85) were found for flour protein fractions (total gliadins, total LMW-GS, and total HMW-GS) and loaf volume. Heritability estimates for gliadin to glutenin ratio (T_Gli:Glu) and SDS unextractable polymeric protein to total polymeric protein ratio (UPP:TPP) were also high (*h*^2^ = 0.9 and 0.69, respectively). Moderate heritability estimates (*h*^2^ = from 0.4 to 0.55) were found for bread flavor, the texture of the crumb, the texture of the crust, and sedimentation volume. Lower heritability estimates (*h*^2^ = from 0.16 to 0.37) were obtained for bread aroma, grain and flour protein concentration, predicted kernel hardness, loaf density, loaf height, dough extensibility score, and total HMW:LMW ratio. Bread aroma, grain and flour protein concentration, predicted kernel hardness, and loaf density were the only quality parameters for which heritability was not significantly different from 0.

**Figure 2:**
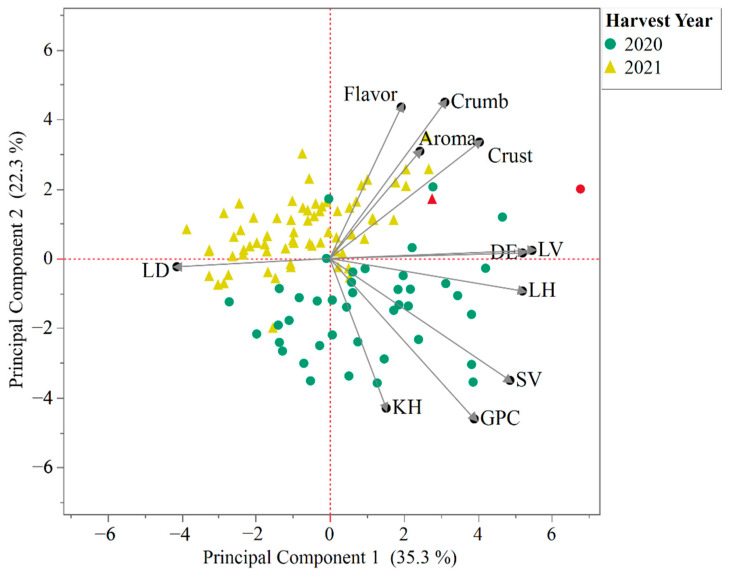
Principal component analysis biplot of 76 wheat genotypes for their quality parameters across the two harvest seasons. The numbers in parentheses on the axis labels refer to the proportion of variance explained by the PC. The red circle and triangle represent the ‘Edison’ cultivar from the harvest years 2020 and 2021, respectively. LD: loaf density; KH: predicted kernel hardness; GPC: grain protein concentration; SV: SDS sedimentation volume; LH: loaf height; DE: dough extensibility score; LV: loaf volume; Crust: texture of the bread crust; Aroma: aroma of the bread; Flavor: flavor of the bread; Crumb: texture of the bread crumb.

Principal components analysis of the 76 wheat genotypes on bread-making quality parameters showed a clear separation of the bread samples according to the harvest year with respect mainly to principal component 2 (PC2; Figure 2). According to the PC loading matrix (Supplemental Table S2), this main differentiation could be explained by sedimentation volume, grain protein concentration, and predicted kernel hardness differences between the two years, and differences in bread sensory parameters scores (flavor, aroma, texture of the crumb, and texture of the crust). The main differentiation across PC1 according to the loading matrix is explained by loaf density (negatively loading PC1) and loaf volume, loaf height, and dough extensibility score (positively loading PC1). Between PC1 and PC2, we were able to explain 57.6% of the variability of the dataset, with PC1 explaining 35.3% of the variability and PC2 explaining 22.3% of the variability. Furthermore, grain samples from the harvest year 2020 showed a higher level of variability, expressed as a wider spread of the data points mainly around principal component 1 (PC1), while samples from 2021 were displayed in a tighter arrangement with respect to this axis.

**Figure 3:**
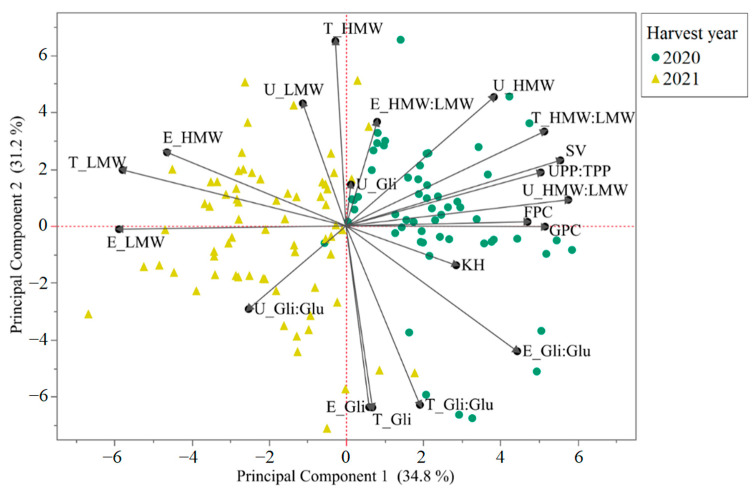
Principal component analysis biplot of 76 wheat genotypes across the two harvest seasons, using the protein fraction parameters obtained through SE-HPLC, grain and flour protein concentration, predicted kernel hardness, and SDS sedimentation volume. The numbers in parenthesis on the axis labels refer to the proportion of variance explained by the PC. “T_”: total SDS extractable and SDS unextractable protein fractions; “U_”: SDS unextractable protein fractions; “E_”: SDS extractable protein fractions; UPP:TPP: SDS unextractable polymeric protein to total polymeric protein ratio; GPC: grain protein concentration; FPC: flour protein concentration; KH: predicted kernel hardness; SV: SDS sedimentation volume.

Principal component analysis of the flour protein fractions, grain and flour protein content, predicted kernel hardness, and SDS sedimentation volume is shown in Figure 3. The separation of the samples between the harvest years is clear, presenting primarily on the PC1 axis. Between the first two PC, we captured 66% of the variability on the dataset. Principal component 1 captured 34.8% of the variation, while PC2 captured 31.2% of the variation in the dataset. According to the loading matrix (Supplemental Table S3), PC1 is loaded positively by SDS sedimentation volume (SV), SDS unextractable polymeric protein to total polymeric protein ratio (UPP:TPP), SDS unextractable HMW-GS to SDS unextractable LMW-GS ratio (U_HMW:LMW), and flour and grain protein concentration. Conversely, PC1 is negatively loaded by SDS extractable LMW-GS (E_LMW), total LWM-GS (T_LMW), and SDS extractable HMW-GS (E_HMW). Regarding PC2, total HMW-GS (T_HMW) is the main positive contributor, while the gliadin fractions (E_Gli, T_Gli, and T_Gli:Glu ratio) contribute negatively to this PC.

**Figure 4:**
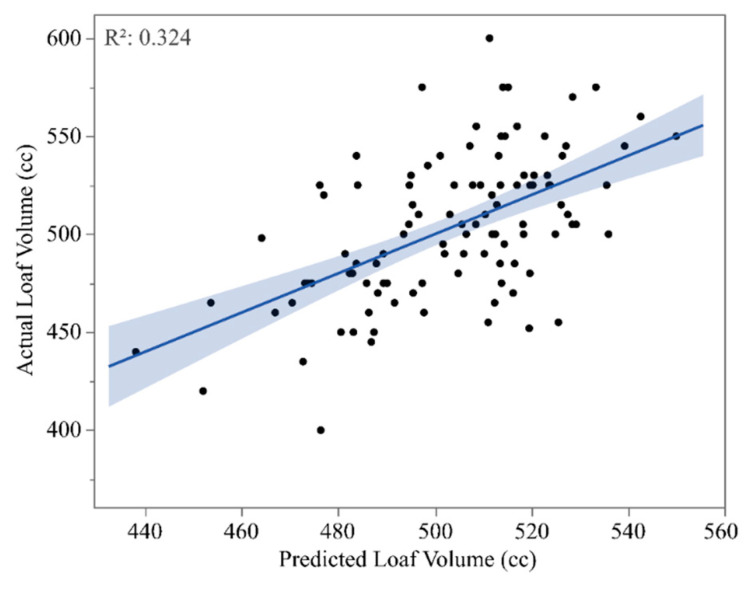
Regression between actual loaf volume and predicted loaf volume from a principal component regression.

Loaf volume prediction through principal component regression (PCR) using the first six PC scores of the protein fractions PCA returned an R^2^ of 0.32 (Figure 4). These first six principal component scores explained almost 97% of the variability of the dataset (Supplemental Table S4).

Pearson’s correlations (Table 2 and Supplemental Table S5) among quality parameters showed that sensorial parameters (i.e., aroma, flavor and texture of the crumb, and the crust) were significantly correlated with each other and significantly correlated with dough functionality parameters. For example, the texture of the crumb and the texture of the crust with respect to sedimentation volume, dough extensibility, loaf height, and loaf volume showed correlation coefficients between 0.30 and 0.45 with associated *p*-values < 0.1. Furthermore, sedimentation volume was significantly correlated (*p*-value < 0.001) with protein concentration (r = 0.51), loaf height (r = 0.57), dough extensibility (r = 0.6), and loaf volume (r = 0.5), and negatively correlated with loaf density (r = −0.33). Flour protein concentration was significantly correlated with grain protein concentration (r = 0.81), SDS sedimentation volume (r = 0.47), and dough extensibility score (r = 0.4), but it was not significantly correlated with loaf volume or loaf density. Regarding protein fractions, loaf volume was negatively correlated with total LMW-GS (r = −0.24) and positively correlated with total HMW:LMW ratio (r = 0.32) and unextractable polymeric protein to total polymeric protein ratio (UPP:TPP; r = 0.53). Interestingly, UPP:TPP was also significantly correlated to the bread sensory parameters texture of the crumb and texture of the crust (r = 0.29 and 0.49, respectively), sedimentation volume (r = 0.63), and loaf height (r = 0.58).

The GWAS results (Table 3) identified 25 significant SNPs associated with dough extensibility (n = 1), loaf height (n = 4), loaf volume (n = 6), texture of the crust (n = 1), flour protein concentration (n = 1), SDS extractable gliadin (‘E_Gliadin’; n = 2), SDS extractable HMW-GS (E_HMW; n = 5), SDS unextractable HMW-GS (U_HMW; n = 4), and SDS unextractable LMW-GS (U_LMW; n = 1). Particularly, loaf volume-associated SNPs had effects of the greatest magnitude (between 9 and 22.5%), while the SNPs associated with the remaining traits had smaller effects (up to 2.5%). These GWAS analyses were conducted using 2-year entry means, as well as individual year entry means and BLUPS. Interestingly, only one SNP was found to be significantly associated with a trait for all environments and models tested (S1B_15439623). This SNP was associated with SDS extractable gliadin with an effect in the range of 1.9 to 2.6%.

**Figure 5:**
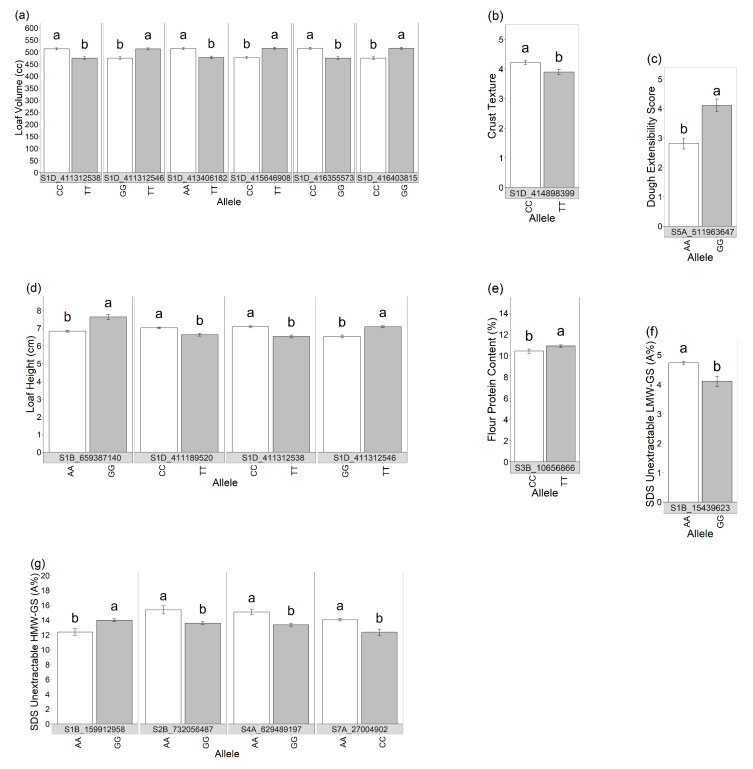

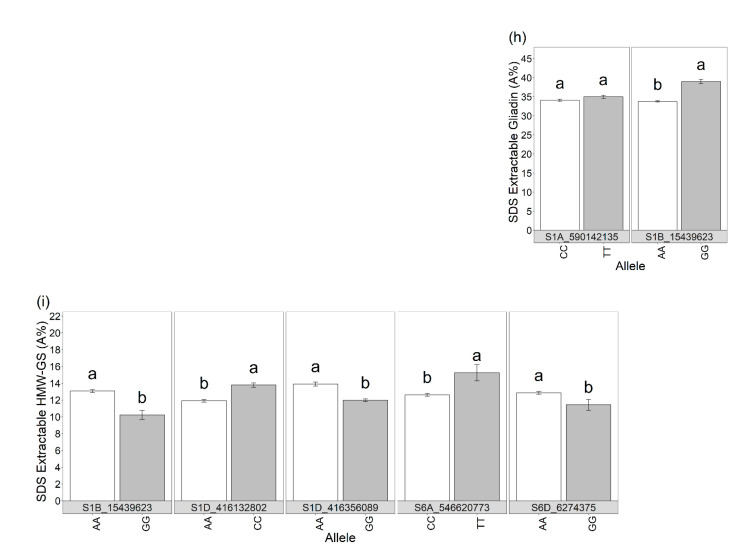
Difference in (**a**) loaf volume, (**b**) crust texture, (**c**) dough extensibility score, (**d**) loaf height, (**e**) flour protein content, (**f**) SDS unextractable LMW-GS, (**g**) SDS unextractable HMW-GS, (**h**) SDS extractable gliadins, and (**i**) SDS extractable HMW-GS due to different allelic form on SNPs identified on average data with GWAS. Vertical lines indicate standard error. Different letters indicate significant differences between allelic forms (α = 0.05).

In addition, we compared the means of the lines for those SNPs that were significantly associated with a trait to determine the robustness of the SNP effect across the array of genotypes through a *t*-test (Figure 5). Our results showed significant differences in all the traits’ means of the lines having different allelic forms for each of the SNPs detected with the GWAS, except for the SNP S1A_590142135 associated with SDS extractable gliadins where the two allelic alternatives did not significantly differ between each other (α = 0.05).

## 4. Discussion

Evaluating the aroma, flavor, and dough characteristics of wheat through bread baking is a high-input and time-consuming activity, mostly carried out during the late stages of the breeding program [35]. The disadvantage of this approach lies in the difficulty of identifying genetic variability late in the breeding program when elite agronomic performers have been selected. Knowing that genetic variation is the main factor determining the success of a breeding program, this strategy is problematic because one might have already discarded promising strong gluten types, for example. To our knowledge, there are few studies evaluating the capability of breeding SRW wheat that could produce high-quality flour for artisan bread-bakers. Such wheat would provide farmers with a specialty crop to grow without the need for extreme diversification of their current production systems.

In this study, we estimated the heritability of easy-to-measure parameters as well as their correlations with the aim of identifying assessments that could be easily repeated in the early stages of the breeding program. Furthermore, we performed a GWAS aiming to find SNPs significantly associated with quality parameters. Previous research has already demonstrated that genotype is the main source of variation regarding flour quality parameters [36]. Additionally, we were able to establish significant genotypic differences among the lines for most of the traits under study, indicating that these are at least partially under genetic control and could be improved through breeding [37,38].

We found that the heritability of flavor and the texture of the crumb and crust assessed with a sensory panel evaluation method was moderate, ranging from 0.4 to 0.5, while the heritability of aroma was not significantly different from zero. Rapp et al. [39] reported similar heritability values for flavor (*h^2^* = 0.56) in a study of spelt bread but higher heritability estimates for aroma (*h^2^* = 0.45) compared to our results. The lack of significant heritability for aroma in our study might be driven by the complexity of aroma determination with the panel evaluation methodology. Furthermore, the standard error of this measurement was the lowest compared to flavor, the texture of the crumb, and the texture of the crust, implying low variability or difficulty in recognizing such variability. However, these four sensorial characteristics were significantly correlated with each other, suggesting that we might be able to indirectly select for aroma by selecting for flavor or texture characteristics.

Regarding dough functionality parameters, we found moderate heritability for SDS sedimentation volume (*h^2^* = 0.53), lower than that reported by [2] for bread wheat (*h^2^* = 0.88) and by [40] for durum wheat (*h^2^* = 0.84), but closer to that reported by [35] for soft winter wheat (*h^2^* = 0.67–0.7). As highlighted by [41], even though SDS sedimentation volume could be regarded independently of weather conditions due to the strong linkage with the genetic pattern of gluten proteins, interactions with nutrient availability can still be observed.

A study on durum wheat reported a heritability estimate for a protein concentration of 0.59 [40], and Rapp et al. [39] reported a heritability estimate of 0.67. While our estimate for protein was not significantly different from zero, none of these studies reported confidence intervals or standard errors associated with their calculations. Furthermore, we observed important differences in weather conditions during harvest seasons 2020 and 2021, with a late frost event around heading in 2020 that reduced the yield potential of the crop and resulted in higher grain protein concentration (2020 protein concentration average = 11.9%, vs. 2021 protein concentration average = 10.1%). On the other hand, harvest season 2021 was excellent for yield performance which could have generated a dilution effect on protein concentration [42].

In addition, the heritability of dough extensibility score (*h^2^* = 0.35) was also lower than that reported in a study for spelt bread (*h^2^* = 0.55) [39] or in a study of bread wheat (*h^2^* = 0.66) [2]. The dough extensibility score is presented in our study as a way of estimating gluten development capacity, which is a function of protein concentration and protein quality [10]. Thus, factors affecting protein concentration (such as the earlier-mentioned weather variability) could also affect dough extensibility. Further support for this idea is found in the significant correlation observed between dough extensibility and protein concentration (*r* = 0.39). Even though only a moderate heritability estimate of dough extensibility was found in our study, high positive correlations were found between this parameter and SDS sedimentation volume (*r* = 0.6) and loaf volume (*r* = 0.53), implying that this low-input and highly repeatable parameter could be useful to estimate dough functionality characteristics.

Even though loaf volume is a particularly important quality determinant, it is one of the most time-consuming and input-demanding tests, so the adaptability and utility in early breeding stages are reduced. Our heritability estimates showed high values for loaf volume (*h^2^* = 0.68), similar to those found by [2] for bread volume (*h^2^* = 0.56) or height/width ratio (*h^2^* = 0.53). In addition, all the identified SNPs had significantly high effect estimates, validated through the t-test, and identified with multiple variations of the model and datasets used to run the GWAS with GAPIT. Unfortunately, we were not able to identify SNPs significantly associated with sensory parameters other than the texture of the crust. We recognize that the number of lines in this study might be small for these types of parameters with low to moderate heritability, which could reflect the intervention of numerous genes with small effects.

In addition to the significant association and strong effect of the six SNPs identified for loaf volume, it is important to highlight that all of them were located on the 1D chromosome. The D genome of hexaploid wheat has been previously and widely associated with quality characteristics [43,44,45,46]. Particularly, Payne [20] showed that bread-making quality was mainly associated with variations at the Glu-D1 locus, with the allelic pairs 1Dx5-1Dy10 (5 + 10 subunits) and 1Dx2-1Dy12 (2 + 12 subunits) detected for high and low bread-baking quality, respectively. Moreover, based on conversations with Noah DeWitt [47] and previous research that identified DNA markers for HMW glutenin subunits [48,49,50,51], we hypothesized that the most significant SNPs we identified could be associated with the Glu-D1 gene.

Regarding the effects of protein composition, our results showed high heritability estimates for all the protein fractions under study, similar to those reported by [52] for spring bread wheat. Considering the effect of protein composition on bread-baking quality, we found that loaf volume was positively correlated with UPP:TPP (*r* = 0.53) and HMW:LMW (*r* = 0.32), with similar results reported by [14]. SDS sedimentation volume was significantly correlated with the HMW:LMW ratio, as well as negatively correlated with total gliadin. Even though other researchers have reported a significant negative correlation between the Gli:Glu ratio and loaf volume [53], we were not able to establish such a relationship in our dataset. In contrast, we found similar positive correlations as those reported by [53] between dough extensibility and loaf volume with HMW:LMW ratio. SDS unextractable polymeric protein fraction tends to show a greater molecular weight compared to the SDS extractable fraction, thus affecting baking properties [14,54,55]. Similarly, we found significant positive correlations between loaf volume and U_Glu (SDS unextractable glutenin) and a significant negative correlation with E_Glu (SDS extractable glutenin) and E_LMW (SDS extractable LMW-GS).

Considering that this research was conducted in the context of a breeding program and that our goal was to identify breeding lines that have desirable baking qualities, after two years of bread baking and evaluation, we selected those lines with the highest mean loaf volume from the initial set of 76 lines. This set of selected lines included 21 genotypes in the top-10 rank for loaf volume (more than one line had the same loaf volume value). If instead of selecting based on loaf volume, we selected based on top-10 values for SDS sedimentation volume, based on the average data, we would have selected 25 lines from which 15 lines were also top-10 loaf volume lines. When selecting genotypes to advance in the breeding program, the breeder would typically select based on one year of data, so it is important to highlight that SDS sedimentation volume from the year 2020 was not highly correlated with the year 2021 (*r* = 0.32), and the breeder could incur an error and select undesirable lines following this approach. To illustrate this, of the 34 top-10 lines in 2020 for sedimentation volume, only 14 became top-10 loaf volume lines in 2021 (41%). In this sense, the UPP:TPP ratio (SDS unextractable polymeric protein to total polymeric protein) seems to be a more reliable parameter based on the high heritability estimate (*h^2^* = 0.7) and correlation coefficient between the year 2020 and year 2021 data (*r* = 0.53). Based on this parameter, if we selected the top-10 lines for UPP:TPP in 2020, we would have selected 12 lines, from which 6 were in the top-10 loaf volume rank in 2021 (50%). However, it must be noted that the UPP:TPP parameter requires vastly more time, energy, and equipment than the SDS sedimentation test, so a breeding program would have to weigh the costs and benefits of this choice.

## 5. Conclusions

Identifying high-quality bread-baking SRW wheat genotypes might represent a challenge, but we were able to select a few parameters that can be used in the early stages of a breeding program that could help to increase the availability of variation and that showed significant heritability estimates. From our results, SDS sedimentation volume, dough extensibility score, and selecting those SNPs highly associated with loaf volume could be easily implemented strategies. Furthermore, knowledge of flour protein composition from the SE-HPLC could significantly improve the information that a breeder has available to make indirect selections for loaf volume, but this technology might not be as easily available as the SDS sedimentation volume test.

Ultimately, our results become more significant if we consider that the amount of variation available in the breeding stage of these lines was very likely limited when the lines were chosen for this study. That said, we also recognize that the number of lines in this study might be small for a GWAS, and we look forward to continuing the investigation of the associated SNPs. Consumers continue to be interested in local foods and working in specialty small grains is emergent in our region as compared to other value chains (e.g., produce). Therefore, developing acceptable bread wheat within a market class traditionally designated for low-protein products has significance for economic diversification on farms and local and regional food systems as a whole.

## Figures and Tables

**Table 1:** Summary of statistics and ANOVA components used to calculate the heritability of the quality parameters evaluated in this study. “T_”: total SDS extractable and SDS unextractable protein fractions; UPP:TPP: SDS unextractable polymeric protein to total polymeric protein ratio. (*) *p*-value < 0.1, (**) *p*-value < 0.05, (***) *p*-value < 0.001, and (ns) not significant. ^†^ Scale from a 1 (dislike very much) to 7 (like very much) with unit intervals. ^‡^ Scale from 1 (lowest resistance to stretching) to 7 (highest resistance to stretching).

Quality Parameter	Units	Entry Means			Mean Squares			
		Min	Max	Mean	Year	Genotype	Genotype × Year	Error
Aroma	1–7 scale ^†^	3.10	5.20	4.0	4.72	1.57 *	1.23	1.24
Flavor	1–7 scale ^†^	3.10	5.20	4.0	10.47	1.75 **	1.06	1.28
Texture: Crumb	1–7 scale ^†^	2.70	5.10	3.70	7.04	2.47 ***	1.11	1.17
Texture: Crust	1–7 scale ^†^	3.20	5.10	4.10	1.58	2.17 ***	1.13	1.07
Grain Protein Concentration	%	9.12	14.64	11.27	12.43	1.67 ***	1.19	0.09
Kernel Hardness	%	7.32	31.12	19.36	40.22	30.20 ***	24.02	2.72
SDS Sedimentation Volume	cm^3^	4.75	15.25	9.25	1548.39	5.71 ***	2.69	0.17
Loaf Volume	cm^3^	400	625	502	3517.66	1875.86 ***		602.08
Loaf Density	gr/cm^3^	0.41	0.74	0.53	6.15 × 10^−7^	0.0021 ^ns^		0.002
Loaf Height	cm	5.4	8.9	6.9	2.73	0.33 **		0.20
Dough extensibility score	1–7 scale ^‡^	1.00	7.00	3.30	35.03	3.31 **		2.15
Flour Protein Concentration	%	8.28	15.07	10.87		0.93 ^ns^		0.76
T_HMW-GS	A%	21.33	31.59	26.46		5.18 ***		0.79
T_LMW-GS	A%	10.21	16.03	13.06		0.98 ***		0.18
T_Gli	A%	35.35	45.14	39.34		5.85 ***		1.18
T_HMW:LMW	-	1.50	2.44	2.04		0.02 *		0.01
T_Gli:Glu	-	0.79	1.36	1.00		0.02 ***		0.002
UPP:TPP	-	0.34	0.59	0.47		0.002 ***		0.001

**Table 2:** Pearson’s correlation coefficients from average data of harvest years 2020 and 2021. LD: loaf density; KH: predicted kernel hardness; GPC: grain protein concentration; SV: SDS: sedimentation volume; LH: loaf height; DE: dough extensibility score; LV: loaf volume; Crust: texture of the bread crust; Aroma: aroma of the bread; Flavor: flavor of the bread; Crumb: texture of the bread crumb; FPC: flour protein concentration; “T_”: total SDS extractable + unextractable fractions; UPP:TPP: SDS unextractable polymeric protein to total polymeric protein ratio. (*) *p*-value < 0.1, (**) *p-*value < 0.05, (***) *p*-value < 0.001, and (ns) not significant.

	Flavor	Crumb	Crust	GPC	KH	SV	LH	DE	LV	LD	FPC	T_HMW	T_LMW	T_Gli	T_HMW: LMW	T_Gli: Glu	UPP:TPP
**Aroma**	0.4 **	0.43 ***	0.35 *	0.15 ^ns^	−0.01 ^ns^	0.39 **	0.28 *	0.51 ***	0.32 *	−0.18 ^ns^	0.16 ^ns^	0.13 ^ns^	−0.01 ^ns^	0.14 ^ns^	0.16 ^ns^	−0.1 ^ns^	0.21 ^ns^
**Flavor**		0.58 ***	0.4 ***	0.04 ^ns^	−0.1 ^ns^	0.36 *	0.31 *	0.34 *	0.26 *	−0.12 ^ns^	−0.01 ^ns^	0.23 ^ns^	0.02 ^ns^	−0.2 ^ns^	0.25 *	−0.17 ^ns^	0.24 ^ns^
**Crumb**			0.66 ***	0.08 ^ns^	−0.16 ^ns^	0.32 *	0.3 *	0.44 ***	0.34 *	−0.37 *	0.12 ^ns^	0.18 ^ns^	−0.02 ^ns^	−0.09 ^ns^	0.22 ^ns^	−0.09 ^ns^	0.29 *
**Crust**				0.18 ^ns^	−0.02 ^ns^	0.45 ***	0.39 **	0.45 ***	0.4 **	−0.35 *	0.17 ^ns^	0.19 ^ns^	−0.12 ^ns^	−0.12 ^ns^	0.35 *	−0.08 ^ns^	0.49 ***
**GPC**					0.5 ***	0.51 ***	0.27 *	0.39 **	0.21 ^ns^	−0.12 ^ns^	0.81 ***	−0.27 *	−0.39 **	0.28 *	0.13 ^ns^	0.32 **	0.23 *
**KH**						0.2 ^ns^	0.07 ^ns^	−0.05 ^ns^	−0.09 ^ns^	0.14 ^ns^	0.31 *	−0.33 *	−0.23 ^ns^	0.32 *	−0.12 ^ns^	0.33 **	0.11 ^ns^
**SV**							0.57 ***	0.6 ***	0.5 ***	−0.33 *	0.47 ***	0.29 *	−0.1 ^ns^	−0.24 ^ns^	0.45 ***	−0.23 ^ns^	0.63 ***
**LH**								0.47 ***	0.66 ***	−0.45 ***	0.27 *	0.29 *	−0.1 ^ns^	−0.24 ^ns^	0.45 ***	−0.21 ^ns^	0.58 ***
**DE**									0.54 ***	−0.46 ***	0.4 **	0.14 ^ns^	−0.11 ^ns^	−0.19 ^ns^	0.41 **	0.16 ^ns^	0.25 *
**LV**										−0.76 ***	0.1 ^ns^	0.06 ^ns^	−0.24 *	−0.07 ^ns^	0.32 *	0.01 ^ns^	0.53 ***
**LD**											−0.08 ^ns^	−0.02 ^ns^	0.15 ^ns^	−0.01 ^ns^	−0.18 ^ns^	−0.02 ^ns^	−0.4 *
**FPC**												−0.08 ^ns^	−0.18 ^ns^	0.12 ^ns^	0.14 ^ns^	0.11 ^ns^	0.06 ^ns^
**T_HMW**													0.63 ***	−0.91 ***	0.49 ***	−0.96 ***	0.1 ^ns^
**T_LMW**														−0.65 ***	−0.38 **	−0.76 ***	−0.34 *
**T_Gli**															−0.36 *	0.97 ***	−0.09 ^ns^
**T_HMW:LMW**																−0.29 *	0.49 ***
**T_Gli:Glu**																	0.01 ^ns^

**Table 3:** Genome-wide association study (GWAS) for 76 SRW wheat lines grown in 2020 (ENV 1), 2021 (ENV 2), average data from 2020 and 2021 seasons (ENV 3), and BLUPS (ENV4), tested under BLINK, FarmCPU, and GLM models using GAPIT. Only SNPs with an effect size higher than 0.1% and a false discovery rate (FDR) adjusted *p*-value smaller than 0.1 are shown in this table. The effect of each SNP is expressed as a percentage of the mean of each trait.

Trait	SNP	Chr	Position	Environment	Model	Effect (%)	FDR Adj. *p*-Value
CRUST TEXTURE	S1D_414898399	1D	414898399	ENV 1 (2020)	FARMCPU	0.35	4.7 × 10^−2^
				ENV 3 (AVERAGE)	BLINK	0.24	9.3 × 10^−3^
LOAF HEIGHT	S1B_659387140	1B	659387140	ENV 3 (AVERAGE)	BLINK	0.26	6.2 × 10^−2^
					FARMCPU	0.21	1.6 × 10^−3^
				ENV 4 (BLUPS)	BLINK	0.15	2.8 × 10^−3^
					FARMCPU	0.12	3.6 × 10^−4^
					GLM	0.19	4.5 × 10^−2^
	S1D_411189520	1D	411189520	ENV 1 (2020)	BLINK	−0.32	6.3 × 10^−5^
					GLM	−0.33	7.2 × 10^−2^
				ENV 3 (AVERAGE)	GLM	−0.21	1.5 × 10^−2^
	S1D_411312538	1D	411312538	ENV 1 (2020)	GLM	−0.33	7.2 × 10^−2^
				ENV 3 (AVERAGE)	GLM	−0.27	4.9 × 10^−3^
				ENV 4 (BLUPS)	GLM	−0.13	4.5 × 10^−2^
	S1D_411312546	1D	411312546	ENV 1 (2020)	GLM	0.33	7.2 × 10^−2^
				ENV 3 (AVERAGE)	BLINK	0.25	5.3 × 10^−6^
					FARMCPU	0.20	8.8 × 10^−6^
					GLM	0.27	4.9 × 10^−3^
				ENV 4 (BLUPS)	BLINK	0.12	1.0 × 10^−4^
					FARMCPU	0.09	3.5 × 10^−4^
					GLM	0.13	4.5 × 10^−2^
LOAF VOLUME	S1D_411312538	1D	411312538	ENV 1 (2020)	FARMCPU	−22.37	1.5 × 10^−2^
				ENV 4 (BLUPS)	FARMCPU	−12.50	7.0 × 10^−2^
	S1D_411312546	1D	411312546	ENV 1 (2020)	FARMCPU	20.21	3.4 × 10^−2^
				ENV 4 (BLUPS)	FARMCPU	12.50	7.0 × 10^−2^
	S1D_413406182	1D	413406182	ENV 1 (2020)	BLINK	−21.08	2.2 × 10^−4^
					FARMCPU	−21.08	1.5 × 10^−2^
	S1D_415646908	1D	415646908	ENV 1 (2020)	FARMCPU	19.75	2.5 × 10^−2^
				ENV 3 (AVERAGE)	BLINK	17.41	2.1 × 10^−4^
					FARMCPU	9.07	3.0 × 10^−2^
				ENV 4 (BLUPS)	FARMCPU	12.41	5.5 × 10^−2^
	S1D_416355573	1D	416355573	ENV 1 (2020)	FARMCPU	−19.31	4.2 × 10^−2^
				ENV 4 (BLUPS)	FARMCPU	−12.94	5.5 × 10^−2^
	S1D_416403815	1D	416403815	ENV 1 (2020)	FARMCPU	22.50	2.2 × 10^−2^
				ENV 4 (BLUPS)	FARMCPU	12.95	5.5 × 10^−2^
DOUGH EXTENSIBILITY	S5A_511963647	5A	511963647	ENV 1 (2020)	BLINK	1.17	3.7 × 10^−6^
					FARMCPU	0.63	6.6 × 10^−2^
				ENV 3 (AVERAGE)	BLINK	0.75	4.4 × 10^−7^
					FARMCPU	0.58	9.9 × 10^−6^
				ENV 4 (BLUPS)	BLINK	0.30	6.9 × 10^−5^
					FARMCPU	0.22	1.6 × 10^−4^
FLOUR PROTEIN	S3B_10656866	3B	10656866	ENV 1 (2020)	BLINK	0.87	1.7 × 10^−3^
					FARMCPU	0.87	4.6 × 10^−2^
E_GLIADIN	S1A_590142135	1A	590142135	ENV 2 (2021)	BLINK	0.87	6.6 × 10^−3^
					FARMCPU	0.70	5.0 × 10^−4^
				ENV 3 (AVERAGE)	FARMCPU	0.55	1.2 × 10^−2^
				ENV 4 (BLUPS)	FARMCPU	0.42	7.1 × 10^−3^
	S1B_15439623	1B	15439623	ENV 1 (2020)	BLINK	2.52	8.6 × 10^−10^
					FARMCPU	2.30	2.4 × 10^−19^
					GLM	2.59	2.4 × 10^−3^
				ENV 2 (2021)	BLINK	2.43	5.8 × 10^−11^
					FARMCPU	2.35	1.6 × 10^−12^
					GLM	2.35	7.2 × 10^−4^
				ENV 3 (AVERAGE)	BLINK	2.50	4.0 × 10^−12^
					FARMCPU	2.10	1.3 × 10^−13^
					GLM	2.46	1.2 × 10^−4^
				ENV 4 (BLUPS)	BLINK	2.05	3.3 × 10^−12^
					FARMCPU	1.91	3.6 × 10^−15^
					GLM	2.02	1.2 × 10^−4^
E_HMW	S1B_15439623	1B	15439623	ENV 1 (2020)	BLINK	−1.07	6.0 × 10^−3^
				ENV 2 (2021)	BLINK	−1.28	8.8 × 10^−6^
					FARMCPU	−1.06	1.1 × 10^−7^
					GLM	−1.58	8.2 × 10^−3^
				ENV 3 (AVERAGE)	BLINK	−1.13	7.5 × 10^−6^
					FARMCPU	−1.23	4.9 × 10^−9^
					GLM	−1.41	5.9 × 10^−3^
				ENV 4 (BLUPS)	BLINK	−0.82	2.8 × 10^−6^
					FARMCPU	0.59	3.7 × 10^−8^
					GLM	−1.07	7.2 × 10^−3^
	S1D_416132802	1D	416132802	ENV 2 (2021)	GLM	1.03	8.2 × 10^−3^
				ENV 3 (AVERAGE)	GLM	0.96	5.4 × 10^−3^
				ENV 4 (BLUPS)	BLINK	0.59	1.0 × 10^−6^
					FARMCPU	−0.81	4.0 × 10^−8^
					GLM	0.71	7.2 × 10^−3^
	S1D_416356089	1D	416356089	ENV 2 (2021)	BLINK	−0.89	8.8 × 10^−6^
					FARMCPU	−1.03	1.6 × 10^−10^
					GLM	−1.11	8.2 × 10^−3^
				ENV 3 (AVERAGE)	BLINK	−0.74	1.1 × 10^−5^
					FARMCPU	−0.61	1.6 × 10^−4^
					GLM	−0.98	5.4 × 10^−3^
				ENV 4 (BLUPS)	GLM	−0.71	7.2 × 10^−3^
	S6A_546620773	6A	546620773	ENV 3 (AVERAGE)	BLINK	1.12	1.5 × 10^−2^
					FARMCPU	0.84	1.0 × 10^−1^
	S6D_6274375	6D	6274375	ENV 2 (2021)	FARMCPU	−1.18	2.0 × 10^−4^
				ENV 4 (BLUPS)	BLINK	−0.97	1.1 × 10^−3^
					FARMCPU	−0.90	3.5 × 10^−3^
U_HMW	S1B_159912958	1B	159912958	ENV 1 (2020)	BLINK	0.96	3.4 × 10^−2^
					FARMCPU	1.23	2.2 × 10^−2^
	S2B_732056487	2B	732056487	ENV 1 (2020)	BLINK	−0.97	3.4 × 10^−2^
					FARMCPU	−1.37	3.7 × 10^−2^
	S4A_629489197	4A	629489197	ENV 3 (AVERAGE)	BLINK	−1.05	1.9 × 10^−4^
				ENV 4 (BLUPS)	BLINK	−0.75	1.1 × 10^−3^
	S7A_27004902	7A	27004902	ENV 3 (AVERAGE)	BLINK	−0.84	3.5 × 10^−3^
				ENV 4 (BLUPS)	BLINK	−0.60	1.2 × 10^−2^
U_LMW	S1B_15439623	1B	15439623	ENV 1 (2020)	BLINK	−0.46	1.4 × 10^−6^
					FARMCPU	−0.44	4.4 × 10^−8^

## Data Availability

The data used to support the findings of this study can be made available by the corresponding author upon request.

## Supplementary Materials

The following supporting information can be downloaded at: https://www.mdpi.com/article/10.3390/foods12132617/s1, Table S1: Precipitation (mm) and maximum, minimum, and average temperatures (°C) for both seasons (2019–2020 and 2020–2021); Table S2: Principal component analysis loading scores matrix; Table S3: Loading score matrix of principal component analysis of the flour protein fractions; Table S4: Eigenvalues of the protein fractions PCA, individual percentage of variability explained by each component, and cumulative percentage; Table S5. Pearson’s correlation coefficient for all the traits under study.

## References

[1] Giraldo P., Benavente E., Manzano-Agugliaro F., Gimenez E. (2019). Worldwide research trends on wheat and barley: A bibliometric comparative analysis. Agronomy.

[2] Longin F., Beck H., Gütler H., Heilig W., Kleinert M., Rapp M., Philipp N., Erban A., Brilhaus D., Mettler-Altmann T. (2020). Aroma and quality of breads baked from old and modern wheat varieties and their prediction from genomic and flour-based metabolite profiles. Food Res. Int..

[3] (2022). USDA—National Agricultual Statistics Service. https://quickstats.nass.usda.gov/.

[4] Rekik F., van Es H., Hernandez-Aguilera J.N., Gómez M.I. (2019). Linking coffee to soil: Can soil health increase coffee cup quality in Colombia?. Soil Sci..

[5] Arnold R.J., Ochoa A., Kerth C.R., Miller R.K., Murray S.C. (2019). Assessing the impact of corn variety and Texas terroir on flavor and alcohol yield in new-make bourbon whiskey. PLoS ONE.

[6] Starr G., Bredie W.L.P., Hansen Å.S. (2013). Sensory profiles of cooked grains from wheat species and varieties. J. Cereal Sci..

[7] Chang C.-Y., Seitz L.M., Chambers E. (1995). Volatile flavor components of breads made from hard red winter wheat and hard white winter wheat. Cereal Chem..

[8] Laidig F., Hüsken A., Rentel D., Piepho H.-P. (2022). Protein use efficiency and stability of baking quality in winter wheat based on the relation of loaf volume and grain protein content. Theor. Appl. Genet..

[9] Starr G., Hansen Å.S., Petersen M.A., Bredie W. (2015). Aroma of wheat porridge and bread-crumb is influenced by the wheat variety. LWT-Food Sci. Technol..

[10] Guttieri M.J., Bowen D., Gannon D., O’Brien K., Souza E. (2001). Solvent retention capacities of irrigated soft white spring wheat flours. Crop Sci..

[11] Wang Y., Zhen S., Luo N., Han C., Lu X., Li X., Xia X., He Z., Yan Y. (2016). Low molecular weight glutenin subunit gene Glu-B3h confers superior dough strength and breadmaking quality in wheat (*Triticum aestivum* L.). Sci. Rep..

[12] Bajgain P., Sallam A.H., Annor G., Conley E., Steffenson B.J., Muehlbauer G.J., Anderson J.A. (2021). Genetic characterization of flour quality and bread-making traits in a spring wheat nested association mapping population. Crop Sci..

[13] Singh N., Donovan R., MacRitchie F. (1990). Use of sonication and SE-HPLC in the study of wheat flour proteins. I. Dissolution of total proteins in the absence of reducing agents. Cereal Chem..

[14] Gupta R., Khan K., Macritchie F. (1993). Biochemical basis of flour properties in bread wheats. I. Effects of variation in the quantity and size distribution of polymeric protein. J. Cereal Sci..

[15] Ohm J.B., Ross A., Ong Y.L., Peterson C. (2006). Using multivariate techniques to predict wheat flour dough and noodle characteristics from size-exclusion HPLC and RVA data. Cereal Chem..

[16] Callejo M.J. (2011). Present situation on the descriptive sensory analysis of bread. J. Sens. Stud..

[17] Duizer L., Walker S. (2016). The application of sensory science to the evaluation of grain-based foods. Encyclopedia of Food Grains.

[18] Herb D., Filichkin T., Fisk S., Helgerson L., Hayes P., Meints B., Jennings R., Monsour R., Tynan S. (2017). Vinkemeier. Effects of barley (*Hordeum vulgare* L.) variety and growing environment on beer flavor. J. Am. Soc. Brew. Chem..

[19] Branlard G., Dardevet M., Saccomano R., Lagoutte F., Gourdon J. (2001). Genetic diversity of wheat storage proteins and bread wheat quality. Euphytica.

[20] Payne P.I. (1987). Genetics of wheat storage proteins and the effect of allelic variation on bread-making quality. Annu. Rev. Plant Physiol..

[21] Shewry P.R., Tatham A.S., Lazzeri P. (1997). Biotechnology of wheat quality. J. Sci. Food Agric..

[22] Soil Survey Staff, N.R.C.S., United States Department of Agriculture (USDA) (2022). Web Soil Survey. http://websoilsurvey.sc.egov.usda.gov/.

[23] Herbek J., Lee C. (2009). A comprehensive guide to wheat management in Kentucky. Cooperative Extension Service.

[24] Fuertes-Mendizábal T., Aizpurua A., González-Moro M.B., Estavillo J.M. (2010). Improving wheat breadmaking quality by splitting the N fertilizer rate. Eur. J. Agron..

[25] Cereals & Grains Association (2000). AACC Approved Methods. Method 56–70: Sodium Dodecyl Sulfate Sedimentation Test for Durum Wheat.

[26] Malalgoda M., Ohm J.-B., Meinhardt S., Simsek S. (2018). Association between gluten protein composition and breadmaking quality characteristics in historical and modern spring wheat. Cereal Chem..

[27] Ohm J.B., Hareland G., Simsek S., Seabourn B. (2009). Size-exclusion HPLC of protein using a narrow-bore column for evaluation of breadmaking quality of hard spring wheat flours. Cereal Chem..

[28] Ohm J.B., Ross A., Peterson C., Ong Y.L. (2008). Relationships of high molecular weight glutenin subunit composition and molecular weight distribution of wheat flour protein with water absorption and color characteristics of noodle dough. Cereal Chem..

[29] Knapp S., Stroup W., Ross W. (1985). Exact confidence intervals for heritability on a progeny mean basis 1. Crop Sci..

[30] Baardseth P., Kvaal K., Lea P., Ellekjaer M., Færgestad E. (2000). The effects of bread making process and wheat quality on French baguettes. J. Cereal Sci..

[31] Martens H., Naes T. (1992). Multivariate Calibration.

[32] Verges V.L., Brown-Guedira G.L., Van Sanford D.A. (2021). Genome-wide association studies combined with genomic selection as a tool to increase Fusarium head blight resistance in wheat. Crop Breed. Genet. Genom..

[33] Poland J.A., Rife T.W. (2012). Genotyping-by-sequencing for plant breeding and genetics. Plant Genome.

[34] Wang J., Zhang Z. (2021). GAPIT Version 3: Boosting power and accuracy for genomic association and prediction. Genom. Proteom. Bioinform..

[35] Knott C.A., Van Sanford D.A., Souza E.J. (2009). Genetic variation and the effectiveness of early-generation selection for soft winter wheat quality and gluten strength. Crop Sci..

[36] Souza E., Martin J., Guttieri M., O’Brien K., Habernicht D., Lanning S., McLean R., Carlson G., Talbert L. (2004). Influence of genotype, environment, and nitrogen management on spring wheat quality. Crop Sci..

[37] Halloran G., Lee J. (1979). Plant nitrogen distribution in wheat cultivars. Aust. J. Agric. Res..

[38] Monaghan J.M., Snape J.W., Chojecki A.J.S., Kettlewell P.S. (2001). The use of grain protein deviation for identifying wheat cultivars with high grain protein concentration and yield. Euphytica.

[39] Rapp M., Beck H., Gütler H., Heilig W., Starck N., Römer P., Cuendet C., Uhlig F., Kurz H., Würschum T. (2017). Spelt: Agronomy, quality, and flavor of its breads from 30 varieties tested across multiple environments. Crop Sci..

[40] Longin C.F.H., Sieber A.-N., Reif J.C. (2013). Combining frost tolerance, high grain yield and good pasta quality in durum wheat. Plant Breed..

[41] Arata A.F., Rogers W.J., Tranquilli G.E., Arrigoni A.C., Rondanini D.P. (2021). Nitrogen–sulfur fertilisation effects on gluten composition and industrial quality in Argentinean bread wheat cultivars differing in apparent sulfur recovery. Crop Pasture Sci..

[42] López-Bellido L., Fuentes M., Castillo J.E., López-Garrido F.J. (1998). Effects of tillage, crop rotation and nitrogen fertilization on wheat-grain quality grown under rainfed Mediterranean conditions. Field Crops Res..

[43] Kaltsikes P., Evans L., Bushuk W. (1968). Durum-type wheat with high bread-making quality. Science.

[44] Kerber E., Tipples K. (1969). Effects of the D genome on milling and baking properties of wheat. Can. J. Plant Sci..

[45] Liu C.Y., Shepherd K.W., Rathjen A.J. (1996). Improvement of durum wheat pastamaking and breadmaking qualities. Cereal Chem..

[46] Shewry P.R., Halford N.G., Tatham A.S. (1992). High molecular weight subunits of wheat glutenin. J. Cereal Sci..

[47] DeWitt N. (2022). Personal communication.

[48] Anderson O., Rausch C., Moullet O., Lagudah E. (2003). The wheat D-genome HMW-glutenin locus: BAC sequencing, gene distribution, and retrotransposon clusters. Funct. Integr. Genom..

[49] Appels R., Eversole K., Stein N., Feuillet C., Keller B., Singh N.K., International Wheat Genome Sequencing Consortium (2018). Shifting the limits in wheat research and breeding using a fully annotated reference genome. Science.

[50] Bietz J., Shepherd K., Wall J. (1975). Single-kernel analysis of glutenin: Use in wheat genetics and breeding. Cereal Chem..

[51] Liu S., Chao S., Anderson J.A. (2008). New DNA markers for high molecular weight glutenin subunits in wheat. Theor. Appl. Genet..

[52] Gómez-Becerra H.F., Abugalieva A., Morgounov A., Abdullayev K., Bekenova L., Yessimbekova M., Sereda G., Shpigun S., Tsygankov V., Zelenskiy Y. (2010). Phenotypic correlations, G× E interactions and broad sense heritability analysis of grain and flour quality characteristics in high latitude spring bread wheats from Kazakhstan and Siberia. Euphytica.

[53] Dhaka V., Khatkar B. (2015). Effects of gliadin/glutenin and HMW-GS/LMW-GS ratio on dough rheological properties and bread-making potential of wheat varieties. J. Food Qual..

[54] Field J.M., Shewry P.R., Miflin B.J. (1983). Solubilisation and characterisation of wheat gluten proteins: Correlations between the amount of aggregated proteins and baking quality. J. Sci. Food Agric..

[55] Shewry P.R., Halford N.G., Belton P.S., Tatham A.S. (2002). The structure and properties of gluten: An elastic protein from wheat grain. Philos. Trans. R. Soc. London. Ser. B Biol. Sci..

