# Supplementary material for: Genetic Variation and Heritability of Sensory and Artisan Bread Traits in a Set of SRW Wheat Breeding Lines
Source: Foods. 2023 Jul 6;12(13):2617. doi: 10.3390/foods12132617 (PMC10340696; doi:10.3390/foods12132617)
Supplement: Supplementary file 1 [file foods-12-02617-s001.zip › foods-2472069-supplementary.pdf]

SUPPLEMENTAL INFORMATION

Table S1. Precipitation (mm) and maximum, minimum, and average temperatures (°C) for both seasons (2019-2020 and 2020-2021). Data retrieved from Kentucky Mesonet https://www.kymesonet.org/

Year	Month	T min (°C)	T max (°C)	T avg (°C)	Precipitation (mm)
2019	October	0.6	34.1	15.1	199.64
2019	November	-10.5	17.5	4.7	144.02
2019	December	-8.6	18.7	5.2	156.97
2020	January	-10.1	22.3	4.1	97.03
2020	February	-9.8	19.1	3.3	138.18
2020	March	-4.6	25.3	10.3	128.52
2020	April	-2.5	25.6	11.3	117.86
2020	May	-1.2	29.3	16.5	130.05
2020	June	10.5	31.4	22.5	100.08
2020	July	17	32.4	25.7	137.92
2020	October	-0.1	26.4	13.8	132.08
2020	November	-2.7	25.4	9.8	56.9
2020	December	-13	18	2	62.74
2021	January	-9.1	18	1.1	114.3
2021	February	-14.2	18.8	-0.4	115.57
2021	March	-4.1	22.6	9.9	129.29
2021	April	-4.7	26	12.2	69.34
2021	May	4	30.9	17	85.09
2021	June	10.1	31.9	22.7	159
2021	July	13.5	32.5	23.9	149.61

Table S2. Principal component analysis loading scores matrix. *LD: Loaf density; KH: Predicted kernel hardness; GPC: Grain protein concentration; SV: SDS Sedimentation volume; LH: Loaf height; DE: Dough extensibility score; LV: Loaf volume; Crust: Texture of the bread crust; Aroma: Aroma of the bread; Flavor: Flavor of the bread; Crumb: Texture of the bread crumb.*

	**PC1**	**PC2**
Aroma	0.36	0.45
Flavor	0.29	0.64
Crumb	0.46	0.66
Crust	0.60	0.49
GPC	0.58	-0.68
KH	0.23	-0.63
SV	0.72	-0.52
LH	0.77	-0.14
DE	0.77	0.02
LV	0.81	0.03
LD	-0.61	-0.04

Table S3. Loading score matrix of principal component analysis of the flour protein fractions. *"T_": total SDS extractable and SDS unextractable protein fractions; "U_": SDS unextractable protein fractions; "E_": SDS extractable protein fractions; UPP:TPP: SDS unextractable polymeric protein to total polymeric protein ratio; GPC: grain protein concentration; FPC: flour protein concentration; KH: predicted kernel hardness; SV: SDS sedimentation volume.*

	**PC1**	**PC2**
GPC	0.77	-0.004
KH	0.43	-0.21
SV	0.83	0.34
FPC	0.71	0.02
E_HMW	-0.70	0.39
U_HMW	0.57	0.68
T_HMW	-0.04	0.97
E_LMW	-0.88	-0.02
U_LMW	-0.17	0.64
T_LMW	-0.87	0.29
E_Gli	0.09	-0.95
U_Gli	0.02	0.22
T_Gli	0.10	-0.96
E_HMW:LMW	0.12	0.55
U_HMW:LMW	0.86	0.14
T_HMW:LMW	0.77	0.50
E_Gli:Glu	0.66	-0.66
U_Gli:Glu	-0.38	-0.44
T_Gli:Glu	0.29	-0.94
UPP:TPP	0.76	0.28

Table S4. Eigenvalues of the protein fractions PCA, individual percentage of variability explained by each component, and cumulative percentage.

PC	Eigenvalue	Percent	Cum. Percent
1	6.95	34.76	34.76
2	6.25	31.24	66.00
3	2.65	13.26	79.26
4	1.67	8.36	87.62
5	1.29	6.46	94.08
6	0.50	2.51	96.58
7	0.32	1.60	98.19
8	0.19	0.97	99.15
9	0.10	0.49	99.64
10	0.04	0.21	99.85
11	0.01	0.05	99.90
12	0.01	0.04	99.94
13	0.01	0.03	99.97
14	0.00	0.02	99.99
15	0.00	0.01	100.00
16	0.00	0.00	100.00
17	0.00	0.00	100.00

Table S5. Pearson's correlation coefficient for all the traits under study.

	Flavor	Crumb	Crust	GPC	KH	SV	LH	DE	LV	LD	LW	FPC	E_HMW	U_HMW	T_HMW	E_LMW	U_LMW	T_LMW	U_Glu	E_Glu	T_Glu	E_Gli	U_Gli	T_Gli	E_HMW: LMW	U_HMW: LMW	T_HMW: LMW	E_Gli: Glu	U_Gli: Glu	T_Gli: Glu	UPP:T PP
Aroma	0.40	0.43	0.35	0.14	0.00	0.39	0.28	0.50	0.32	-0.18	-0.37	0.15	-0.08	0.23	0.13	-0.13	0.20	0.00	0.23	-0.10	0.09	-0.16	0.12	-0.14	0.05	0.09	0.16	0.00	-0.12	-0.10	0.21
Flavor		0.58	0.40	0.04	-0.10	0.35	0.30	0.34	0.25	-0.12	0.00	-0.01	-0.02	0.28	0.22	-0.18	0.31	0.02	0.30	-0.08	0.17	-0.23	0.19	-0.20	0.18	0.02	0.24	-0.05	-0.14	-0.17	0.23
Crumb			0.66	0.08	-0.16	0.32	0.30	0.44	0.34	-0.37	-0.24	0.12	-0.08	0.29	0.18	-0.24	0.34	-0.02	0.31	-0.14	0.13	-0.14	0.27	-0.09	0.15	0.00	0.22	0.03	-0.10	-0.09	0.28
Crust				0.18	-0.02	0.45	0.38	0.45	0.39	-0.35	-0.09	0.16	-0.26	0.47	0.18	-0.38	0.38	-0.12	0.47	-0.32	0.09	-0.16	0.25	-0.12	0.04	0.23	0.35	0.14	-0.29	-0.08	0.49
GPC					0.50	0.51	0.27	0.39	0.21	-0.12	0.03	0.80	-0.37	0.04	-0.27	-0.33	-0.18	-0.39	-0.01	-0.38	-0.34	0.26	-0.01	0.28	-0.18	0.33	0.13	0.39	0.00	0.32	0.23
KH						0.19	0.07	-0.05	-0.09	0.13	0.12	0.31	-0.32	-0.08	-0.33	-0.13	-0.21	-0.23	-0.11	-0.27	-0.33	0.32	-0.14	0.32	-0.34	0.20	-0.12	0.35	0.00	0.33	0.10
SV							0.57	0.60	0.50	-0.33	0.03	0.47	-0.31	0.63	0.28	-0.47	0.46	-0.16	0.62	-0.39	0.15	-0.35	0.33	-0.31	0.08	0.35	0.51	0.07	-0.40	-0.23	0.63
LH								0.46	0.66	-0.45	0.14	0.26	-0.28	0.60	0.28	-0.37	0.41	-0.10	0.58	-0.33	0.17	-0.28	0.31	-0.24	0.02	0.38	0.45	0.07	-0.37	-0.21	0.57
DE									0.53	-0.46	-0.11	0.39	-0.02	0.29	0.24	-0.27	0.23	-0.11	0.29	-0.11	0.14	-0.22	0.21	-0.19	0.29	0.17	0.41	-0.05	-0.15	-0.16	0.25
LV										-0.76	-0.12	0.10	-0.35	0.40	0.05	-0.50	0.36	-0.24	0.41	-0.43	-0.05	-0.12	0.29	-0.07	0.04	0.16	0.32	0.22	-0.21	0.00	0.52
LD											0.35	-0.08	0.30	-0.31	-0.02	0.30	-0.22	0.14	-0.30	0.32	0.04	0.03	-0.19	-0.01	0.11	-0.20	-0.18	-0.19	0.19	-0.02	-0.40
LW												-0.12	-0.04	0.20	0.14	-0.16	0.15	-0.06	0.20	-0.08	0.08	-0.10	0.03	-0.11	0.10	0.08	0.20	0.00	-0.20	-0.10	0.17
FPC													-0.12	0.03	-0.08	-0.11	-0.16	-0.18	-0.02	-0.13	-0.12	0.08	0.15	0.11	-0.05	0.28	0.14	0.10	0.17	0.11	0.06
E_HMW														-0.29	0.58	0.73	0.02	0.65	-0.22	0.97	0.66	-0.46	-0.07	-0.51	0.67	-0.49	-0.05	-0.89	0.20	-0.62	-0.75
U_HMW															0.62	-0.37	0.76	0.10	0.99	-0.34	0.49	-0.63	0.48	-0.57	0.00	0.46	0.61	-0.08	-0.66	-0.53	0.83
T_HMW																0.28	0.67	0.62	0.66	0.51	0.96	-0.91	0.35	-0.91	0.55	-0.01	0.48	-0.80	-0.40	-0.96	0.10
E_LMW																	-0.10	0.83	-0.32	0.88	0.51	-0.26	-0.15	-0.32	-0.03	-0.46	-0.57	-0.71	0.23	-0.44	-0.73
U_LMW																		0.47	0.86	-0.02	0.66	-0.74	0.66	-0.66	0.15	-0.22	0.23	-0.35	-0.35	-0.65	0.55
T_LMW																			0.20	0.76	0.82	-0.64	0.23	-0.65	0.06	-0.53	-0.38	-0.83	0.01	-0.76	-0.34
U_Glu																				-0.27	0.56	-0.68	0.55	-0.62	0.04	0.31	0.55	-0.15	-0.61	-0.58	0.80
E_Glu																					0.65	-0.42	-0.10	-0.48	0.46	-0.51	-0.24	-0.89	0.23	-0.59	-0.79
T_Glu																						-0.90	0.34	-0.91	0.43	-0.19	0.22	-0.89	-0.29	-0.98	-0.05
E_Gli																							-0.47	0.98	-0.42	0.09	-0.36	0.78	0.31	0.96	-0.17
U_Gli																								-0.29	0.08	-0.18	0.14	-0.17	0.32	-0.32	0.40
T_Gli																									-0.44	0.06	-0.36	0.81	0.41	0.97	-0.09
E_HMW:LMW																										-0.19	0.56	-0.54	0.03	-0.44	-0.28
U_HMW:LMW																											0.63	0.38	-0.52	0.12	0.52
T_HMW:LMW																												-0.03	-0.49	-0.29	0.49
E_Gli:Glu																													0.00	0.88	0.46
U_Gli:Glu																														0.34	-0.54
T_Gli:Glu																															0.00
